# Cross-sectional analysis of feline gut microbiota reveals differences across age-defined groups under varying environments

**DOI:** 10.3389/fvets.2026.1775401

**Published:** 2026-05-12

**Authors:** Yan Wang, Zehui Chen, Cong Hua, Junfu Mao, Wenjing Geng, Xiaobo Feng, Shaotang Ye, Shanshan Song, Huanan Wang, Xiaodu Wang, Lin Lin

**Affiliations:** 1 MetaHealth Technology (Hangzhou) Co., Ltd., Hangzhou, China; 2 College of Animal Science and Technology and College of Veterinary Medicine, Zhejiang A&F University, Hangzhou, China; 3 New Ruipeng Pet Healthcare Group, Beijing, China; 4 Mei Lian Zhong He Veterinary Referral Center, Beijing, China; 5 Center for Veterinary Sciences, Veterinary Teaching Hospital, Zhejiang University, Hangzhou, China; 6 Department of Veterinary Medicine, College of Animal Sciences, Zhejiang University, Hangzhou, China

**Keywords:** 16S rRNA gene sequencing, age, cross-sectional study, domestic cats, gut microbiota

## Abstract

The gut microbiome plays a critical role in host health; however, its variation across age groups in domestic cats (*Felis catus*) remains unclear. This study characterized differences in the feline gut microbiota using 16S rRNA gene sequencing in 83 cats across five age-defined groups under varying environments: Pre-weaning (1.5 months, *n* = 16), Early kitten (3 months, *n* = 16), Late kitten (6–10 months, *n* = 15), Young adult (2 years, *n* = 20), and Mature adult (7–10 years, *n* = 16). Significant differences in microbial diversity and composition were observed across groups. Alpha-diversity was lowest in Pre-weaning kittens, peaking in Young adults, and declining in Mature adults. Beta-diversity revealed distinct clustering among groups (PERMANOVA, *R*^2^ = 0.33, *p* = 0.001). Sensitivity analysis excluding the heterogeneous Mature adult group showed consistent patterns and increased the explanatory power of age (*R*^2^ = 0.48). At the taxonomic level, Pre-weaning microbiota were enriched in Proteobacteria, particularly *Escherichia-Shigella*. Following weaning, the relative abundance of *Negativibacillus* increased, whereas *Lactobacillus* decreased. *Bifidobacterium* was more abundant in kitten stages, while *Faecalibacterium* exhibited higher abundance in Young adults. These patterns were generally consistent in sensitivity analyses. In contrast, the Mature adult group exhibited reduced *Prevotella* and increased *Escherichia-Shigella* relative to Young adults. Overall, this cross-sectional study identified compositional differences in the feline gut microbiota across age-defined groups under varying environments. These findings should be interpreted as group-level associations rather than independent age effects. Further controlled and longitudinal studies are needed to disentangle these effects.

## 1 Introduction

The gut microbiota, composed of trillions of microorganisms residing in the gastrointestinal tracts of mammals, including cats, plays a pivotal role in maintaining host physiological processes and overall health. The dynamic interaction between the gut microbiota and its host regulates many functions, encompassing nutrient metabolism, immune system modulation, and bidirectional communication with the host (1). Substantial evidence has linked gut microbiota dysbiosis to the pathogenesis of numerous diseases, including inflammatory disorders, metabolic syndromes, allergic reactions, and neurobehavioral developmental abnormalities (2). Consequently, preserving gut microbial homeostasis is fundamental to safeguarding host health throughout the lifespan.

The composition of the gut microbiota is affected by several intrinsic (genetics, age, sex) and extrinsic (environment, physiological state, antibiotic therapy, health and nutrition) factors (3). Among these, age is a significant factor associated with shifts in microbial composition, with gut microbiota profiles often exhibiting distinct characteristics across different life stages—particularly during the growth and senior or geriatric years. Substantial evidence from human studies supports this concept. The composition of the human gut microbiota has been observed to change during an individual’s life (4). A Japanese cross-sectional study (ages 0–104) investigating sequential changes in the gut microbiota of subjects from newborns to centenarians found that Actinobacteria abundance declines significantly post-weaning and continues to decrease with advancing age, whereas Bacteroidota and Proteobacteria exhibit a characteristic increase from the age of 70 years onward. Furthermore, gut microbial diversity rises following weaning, increases sequentially until the early twenties, remains stable throughout adulthood, and then increases again in the elderly stage until the centenarian stage (5). Similarly, Mancabelli et al. demonstrated that young children harbor a unique microbiota with low diversity and distinct taxa, which transitions to a more diverse adult-like community during adolescence (6). With advancing age toward senescence, beneficial gut microbial taxa decline, while inflammation-associated bacteria accumulate. This age-related gut microbiota dysbiosis enhances intestinal permeability and activates the immune system, ultimately contributing to chronic low-grade inflammation, frailty, and increased morbidity in older individuals (7–9).

In the field of feline research, a study on the dynamic development of gut microbiota during and after weaning in kittens revealed significant changes in the intestinal flora post-weaning. On day 4 post-weaning, the abundance of beneficial bacteria including *Bacteroides vulgatus*, *Fusobacterium nucleatum*, *Anaerostipes caccae*, and *Butyricicoccaceae* decreased. However, by day 30, beneficial bacteria such as *Candidatus Arthromitus*, *Holdemanella*, and *Bifidobacterium* significantly increased (10). Masuoka et al. employed a culture-based method to report the intestinal microbiota composition of cats in five age groups (pre-weaning, weaning, young, aged and senior). They found that the feline intestinal microbiota changes with age, and these changes differ from those in humans and dogs. Enterococci, rather than bifidobacteria or lactobacilli, seem to be major lactic-acid-producing bacteria in cats (11). However, culture-based methods have limitations in comprehensively capturing the full diversity and complexity of the microbiota, and the detailed changes across the continuous age gradient in cats remain unclear.

Advances in 16S rRNA gene sequencing technology have revolutionized the study of gut microbiomes, enabling high-throughput, culture-independent analysis of microbial communities. In this study, we utilized this technology to characterize the composition and structure of the feline gut microbiota across five age-defined groups, ranging from Pre-weaning to Mature adult cats. This study aims to describe differences in the feline gut microbiota across age-defined groups under varying environmental conditions. We further acknowledge that these patterns may reflect the combined influences of host development, housing environments, and dietary variation. These findings provide a descriptive framework for future studies designed to disentangle these factors under controlled conditions.

## 2 Materials and methods

### 2.1 Animals

In this study, fresh fecal samples were collected from 83 clinically healthy cats representing five distinct age groups: Pre-weaning (1.5 months, *n* = 16), Early kitten (3 months, *n* = 16), Late kitten (6–10 months, *n* = 15), Young adult (2 years, *n* = 20), and Mature adult (7–10 years, *n* = 16). Age groups were defined in accordance with the 2021 AAHA/AAFP Feline Life Stage Guidelines (12) and are presented in Table 1. Cats in the Pre-weaning, Early kitten, Late kitten, and Young adult groups were sourced from several catteries in Shandong, China, where they were individually housed and fed a standardized commercial dry baked diet (crude protein: 34%, crude fat: 19.8%, ash: 8.2%, moisture: 3.9%) without additional treats or supplements. They were provided with clean water ad libitum and kept in a controlled environment with regular feeding, play, and resting routines. Mature adult cats were client-owned pets undergoing routine physical examinations at veterinary hospitals. These cats were housed in their respective home environments and fed primarily varied commercially available balanced dry diets selected by their owners; no therapeutic or prescription diets were administered. All cats were clinically healthy at the time of sampling, with no signs of gastrointestinal or systemic disease. Exclusion criteria included any disease or medication that might affect gut microbiota: administration of antibiotics, NSAIDs, corticosteroids, prebiotics, probiotics, or anthelmintics within 4 weeks before sampling; presence of chronic systemic disease or long-term medication; dietary changes within 2 weeks before sampling; and abnormal feces or gastrointestinal signs.

**Table 1:** Summary of cat information across age groups.

Age category	Total	Age (mean ± SD)	Sex (M/F)	Neutered (*n*, %)	Body weight (kg, mean ± SD)	Breed distribution (*n*)
Pre-weaning	16	45.8 ± 8.6 days	8/8	0 (0%)	0.78 **±** 0.14	Ragdolls (4), British shorthair (9), Maine Coon (2), Persian (1)
Early kitten	16	3.0 ± 0 months	7/9	0 (0%)	1.15 **±** 0.39	British shorthair (10), American shorthair (1), Ragdolls (5)
Late kitten	15	8.6 ± 1.7 months	8/7	0 (0%)	3.66 ± 1.08	American shorthair (7), Chinese domestic (3), British shorthair (4), Ragdolls (1)
Young adult	20	2.0 ± 0 years	10/10	0 (0%)	4.58 ± 0.74	British shorthair (14), American shorthair (3), Chinese domestic (1), Siamese (1), Ragdolls (1)
Mature adult	16	7.89 ± 0.89 years	7/9	13 (81.25%)	5.34 ± 1.49	Chinese domestic (9), British shorthair (2), American shorthair (1), Siamese (1), Scottish Fold (2), Ragdolls (1)

### 2.2 Collection of fecal samples

Fresh fecal samples were collected immediately after defecation to minimize environmental contamination. For cats housed in catteries, sample collection was performed by trained cattery staff. All samples were immediately placed in specialized preservation solution (MetaHealth Technology (Hangzhou) Co., Ltd., Hangzhou, China) and transported at ambient temperature to the laboratory. Fecal samples from Mature adult (client-owned) cats were collected by veterinary clinical personnel during routine examinations at veterinary hospitals and subsequently transported to the laboratory under identical conditions. This study was reviewed and approved by Animal Care and Use Committee of Zhejiang A&F University.

### 2.3 DNA extraction

Total genomic DNA was extracted from fecal samples using the GHFDE100 DNA isolation kit (Zhejiang Hangzhou Equipment Preparation: 20190952) in accordance with the manufacturer’s instructions. Extracted DNA was stored at −20 °C until further analysis. The quantity and quality of extracted DNAs were measured using a NanoDrop ND-1000 spectrophotometer (Thermo Fisher Scientific, Waltham, MA, United States) and agarose gel electrophoresis, respectively.

### 2.4 16S rDNA amplicon sequencing

The hypervariable V4 region of the bacterial 16S rRNA gene was amplified using primers 515F (5′-GTGCCAGCMGCCGCGGTAA-3′) and 806R (5′-GGACTACHVGGGTWTCTAAT-3′). Sample-specific paired-end 6-bp barcodes were incorporated into the TrueSeq adaptors for multiplex sequencing. The PCR reactions contained 25 μL Phusion High-Fidelity PCR Master Mix (2×), 3 μL (10 μM) of each primer, 10 μL DNA template, 3 μL DMSO and 6 μL ddH2O. Thermal cycling consisted of initial denaturation at 98 °C for 30 s, followed by 25 cycles consisting of denaturation at 98 °C for 15 s, annealing at 58 °C for 15 s, and extension at 72 °C for 15 s, with a final extension of 1 min at 72 °C. Negative controls (no template) were included in each PCR run. PCR amplicons were purified using Agencourt AMPure XP Beads (Beckman Coulter, Indianapolis, IN) and quantified using the PicoGreen dsDNA Assay Kit (Invitrogen, Carlsbad, CA, United States). After the individual quantification step, amplicons were pooled in equal amounts and paired-end sequencing (2 × 150 bp) was performed on the DNBSEQ-G99 platform (MGI Tech Co., Ltd., Shenzhen, China).

### 2.5 Data statistical analysis

Quality filtering of raw paired-end reads was conducted using Vsearch v2.22.1 under predefined filtering criteria to retain high-quality clean reads (13). The filtered reads were subsequently aligned to the SILVA reference database with the UCHIME algorithm for chimera detection and elimination, yielding the final effective tags. Amplicon sequence variants (ASVs) were generated from effective tags using the UNOISE2 denoising algorithm (14, 15). To reduce noise from low-abundance taxa, ASVs with a mean relative abundance < 0.01% across all samples were removed prior to downstream analysis.

The alpha-diversity indices (Chao1 richness, Shannon diversity) were calculated using QIIME2 v2024.10 from the ASV table normalized using the total sum scaling (TSS) method and standardized to a total abundance of 100,000 reads per sample. Group-level differences in alpha-diversity were tested via the Kruskal-Wallis test, followed by Dunn’s *post-hoc* test for pairwise comparisons (FDR-adjusted *p*-value < 0.05). Rank-abundance curves were generated at the ASV level using R (v3.6.3) to visualize taxonomic richness and evenness across different age groups. For Beta-diversity, weighted UniFrac distances and Bray-Curtis dissimilarities were calculated and visualized via principal coordinate analysis (PCoA) (16–18). The significance of differentiation of microbiota structure among groups was assessed by PERMANOVA (Permutational multivariate analysis of variance) using the R package “vegan.” To assess the robustness of group-associated microbiota differences against structural confounding (e.g., diet and housing), a sensitivity analysis was performed by excluding the “Mature adult” group, which represented the primary source of dietary and environmental heterogeneity. This restriction allowed the analysis to focus on a more controlled group with relatively standardized housing conditions and diet. A covariate-adjusted PERMANOVA was not pursued, as key covariates (environment, diet, and neutering status) exhibited minimal variance within the remaining groups after exclusion of the Mature adult group, thereby limiting the interpretability of multivariable modeling.

Differentially abundant taxa across age-defined groups were identified using the ANCOM-BC2 method implemented in the R package ANCOMBC. Taxa with prevalence <20% across all samples were filtered prior to analysis. *p*-values were adjusted using the Benjamini–Hochberg false discovery rate (FDR), with *q* < 0.05 considered statistically significant. *Post-hoc* pairwise comparisons were conducted to assess differences between specific age groups using log-fold change (LFC) as the effect size. A prevalence cutoff of 10% within each group was applied in the ANCOM-BC2 analysis. In addition, an internal prevalence cutoff of 10% was applied within the ANCOM-BC2 model, and structural zeros were not considered in the analysis. Compared with traditional differential abundance methods, ANCOM-BC2 accounts for compositional bias and sampling fraction variation, providing more robust estimates of differentially abundant taxa. Similarly, to verify the stability of specific taxonomic shifts against structural confounding, the ANCOM-BC2 differential abundance analysis was re-performed on this restricted sub-group (excluding the Mature adult group).

Potential KEGG functional profiles were predicted from 16S rRNA gene sequencing data using PICRUSt2 (v2.3.0-b) with its default reference database. To account for compositional bias, predicted functional profiles were normalized using the cumulative sum scaling (CSS) method. Differences in predicted KEGG pathways across age-defined groups were assessed using the `metagenomeSeq` R package. Associations between bacterial genera and predicted KEGG pathways were evaluated using Spearman’s rank correlation analysis, and significant correlations (*p* < 0.05) were visualized as heatmaps. As these functional profiles are inferred from 16S rRNA gene data rather than directly measured, all analyses should be interpreted as predictive and hypothesis-generating.

## 3 Results

### 3.1 Quality control and ASVs analysis

**Figure 1:**
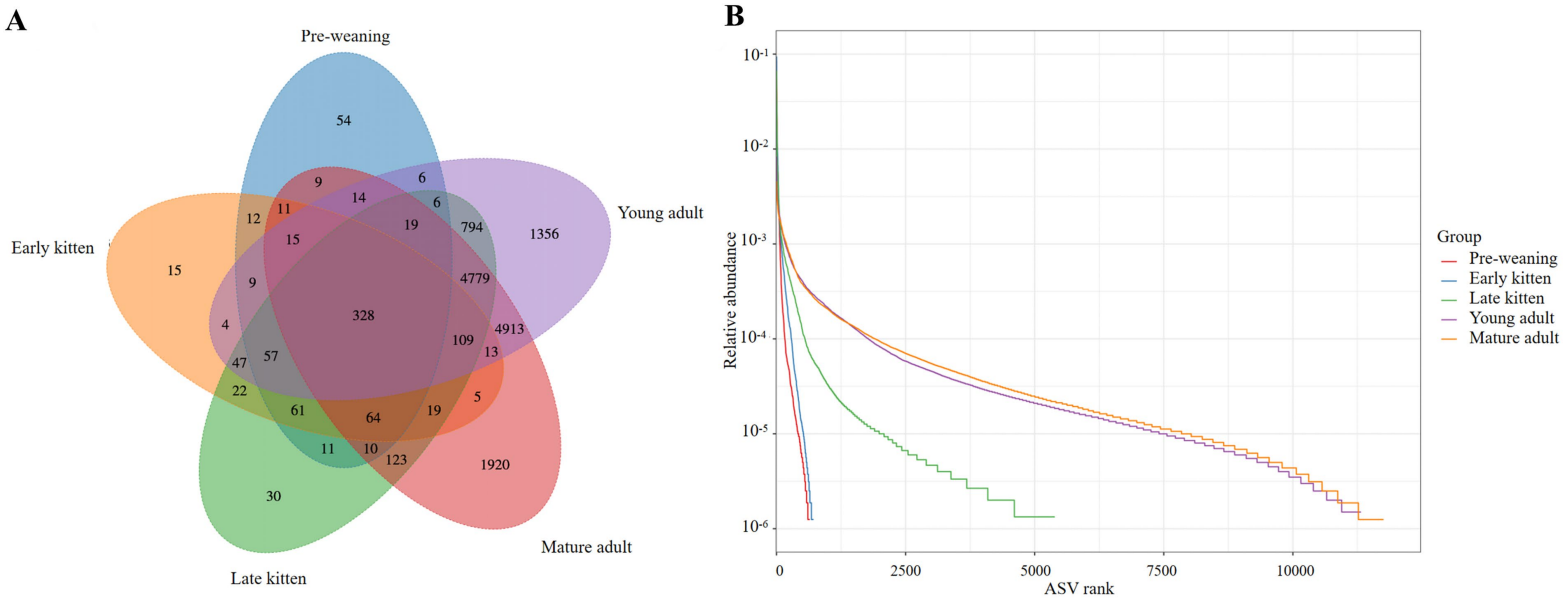
Venn diagram **(A)** and the rank–abundance curves **(B)**. There were 328 shared ASVs among the five age-defined groups, each group also had its corresponding unique ASVs. Rank-abundance curves revealed the bacterial community richness and evenness. Horizontally, curve width reflects species richness, with wider ranges indicating higher richness. Vertically, curve smoothness demonstrates species evenness, where flatter curves represent a more uniform species distribution.

A total of 6,728,665 effective reads were obtained from the 9,782,968 raw reads, with an average of 81,068 ± 19,283 reads per sample. The UNOISE2 algorithm was employed for denoising to generate amplicon sequence variants (ASVs) from the effective tags. As shown in Figure 1, a total of 328 shared ASVs were identified across the five groups (Pre-weaning, Early kitten, Late kitten, Young adult, Mature adult); however, each group also had its corresponding unique ASVs. The Venn diagram revealed that the Young adult and Mature adult groups exhibited the highest numbers of unique ASVs (1,356 and 1,920, respectively) among all groups, suggesting more specialized microbial communities in these stages. Rank-abundance curves further indicated higher gut microbial richness and evenness in the Young adult and Mature adult groups compared to the Pre-weaning, Early kitten, and Late kitten groups, as evidenced by their longer curve extensions (reflecting higher ASV richness) and flatter slopes (indicating more uniform ASV distribution).

### 3.2 Variations in microbial diversity across age-defined groups

**Figure 2:**
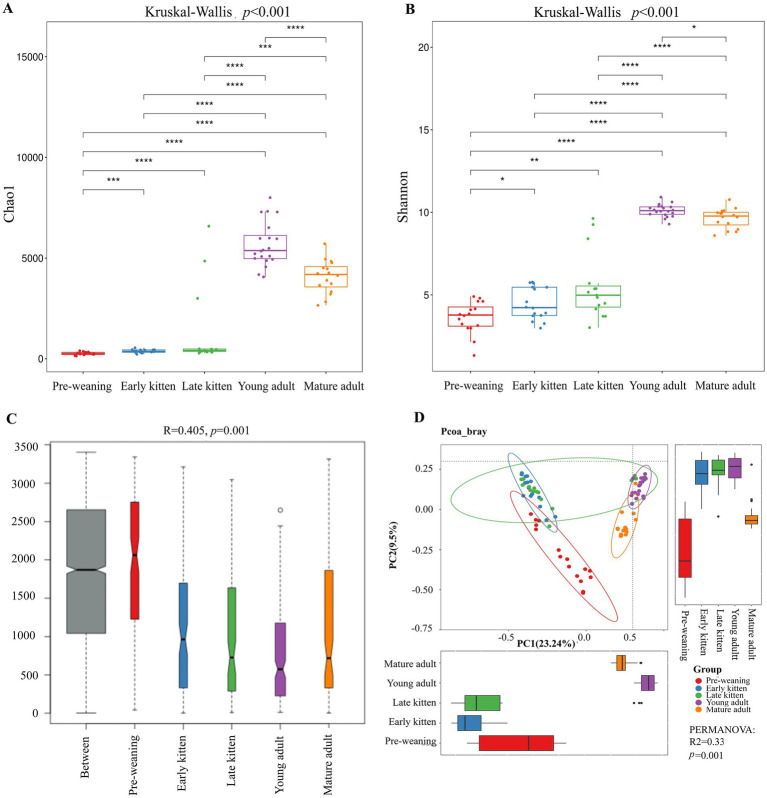
Diversity analysis of samples in the five age-defined groups. **(A)** Chao1 and **(B)** Shannon indices represent microbial richness and evenness, respectively, indicating differences in microbial alpha-diversity among samples. Differences across groups were tested using the Kruskal-Wallis test (overall *p* < 0.001), followed by Dunn’s *post-hoc* test with FDR correction for pairwise comparisons (*q < 0.05, **q < 0.01, ***q < 0.001, ****q < 0.0001). **(C)** Analysis of similarities (ANOSIM) based on weighted UniFrac distances, showing the distribution of between-group distances and within-group distances for each defined stage (*R* = 0.405, *p* = 0.001). **(D)** Principal Coordinate Analysis (PCoA) based on Bray-Curtis dissimilarities, reflecting Beta-diversity differences, with statistical significance confirmed by PERMANOVA (*R*^2^ = 0.33, *p* = 0.001).

Microbial alpha-diversity varied significantly among the five age-defined groups (*p* < 0.001 for both Chao1 and Shannon indices, Figures 2A,B). In the full dataset, Dunn’s *post-hoc* test showed that Pre-weaning kittens exhibited the lowest alpha-diversity compared with all other groups (*p* < 0.05), while no significant differences were detected between Early and Late kitten stages. Alpha-diversity reached its peak in Young adults and declined in the Mature adult stage (*p* < 0.05). To account for potential environmental confounding, a sensitivity analysis was conducted by excluding the Mature adult group (Supplementary Figure S1). This analysis confirmed a consistent increase in alpha-diversity from Pre-weaning to Young adulthood; however, the difference in the Shannon index between the Pre-weaning and Early kitten groups was reduced and did not reach statistical significance within the restricted sub-group.

Beta-diversity analysis further revealed distinct clustering of microbiota structure among the groups (PERMANOVA on Bray-Curtis, *R*^2^ = 0.33, *p* = 0.001, Figure 2D). This was supported by Analysis of Similarities (ANOSIM) based on weighted UniFrac distances, which showed that inter-group dissimilarity was significantly greater than intra-group variability (*R* = 0.405, *p* = 0.001, Figure 2C). Consistent patterns were observed in the sensitivity analysis, and exclusion of the Mature adult group increased the explanatory power (*R*^2^) of age-defined stages from 0.33 to 0.48 (PERMANOVA, *p* = 0.001, Supplementary Figure S2). These results indicate that group-level differences in community structure were more clearly resolved within the restricted group. Together with ASV distribution and rank-abundance data, these results indicate significant structural variation in the feline gut microbiota across age-defined groups.

### 3.3 Variations in core phyla across age-defined groups

**Figure 3:**
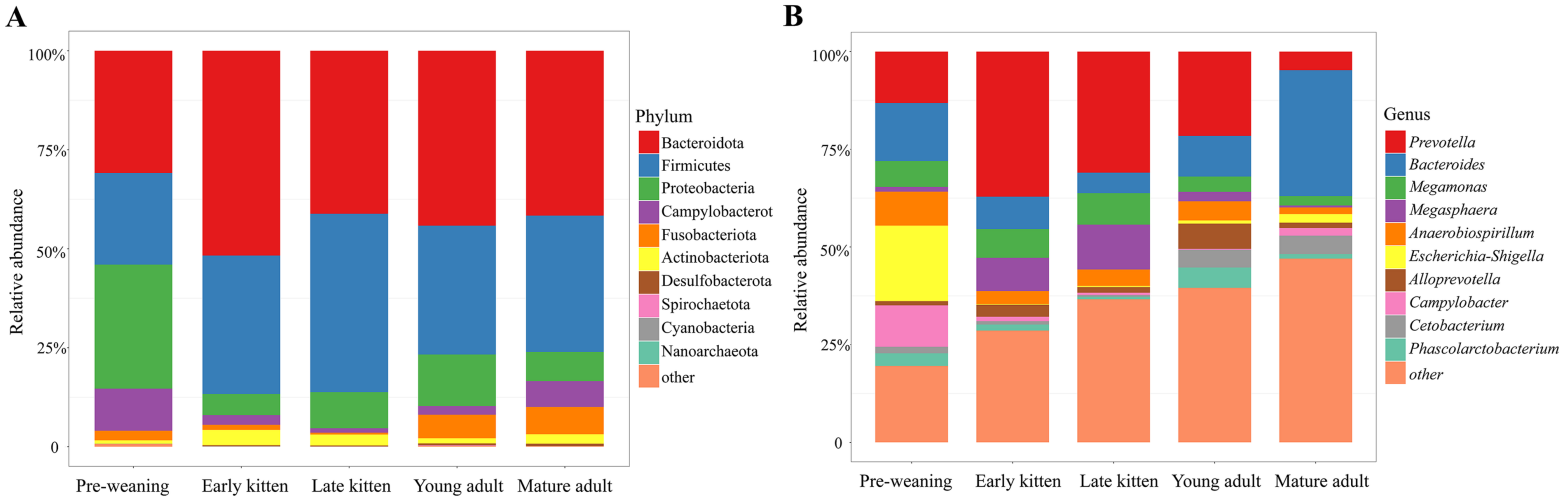
Distribution characteristics of the gut microbiota across age-defined groups of cats: **(A)** Relative abundance of bacteria at the phylum level and **(B)** relative abundance of bacteria at the genus level.

The taxonomic composition of the feline gut microbial communities was analyzed at the phylum level. Across all sequenced samples, the dominant bacterial phyla were Bacteroidota, Firmicutes, Proteobacteria, Campylobacterota, Fusobacteriota, and Actinobacteriota (Figure 3A; Supplementary Table S1).

**Figure 4:**
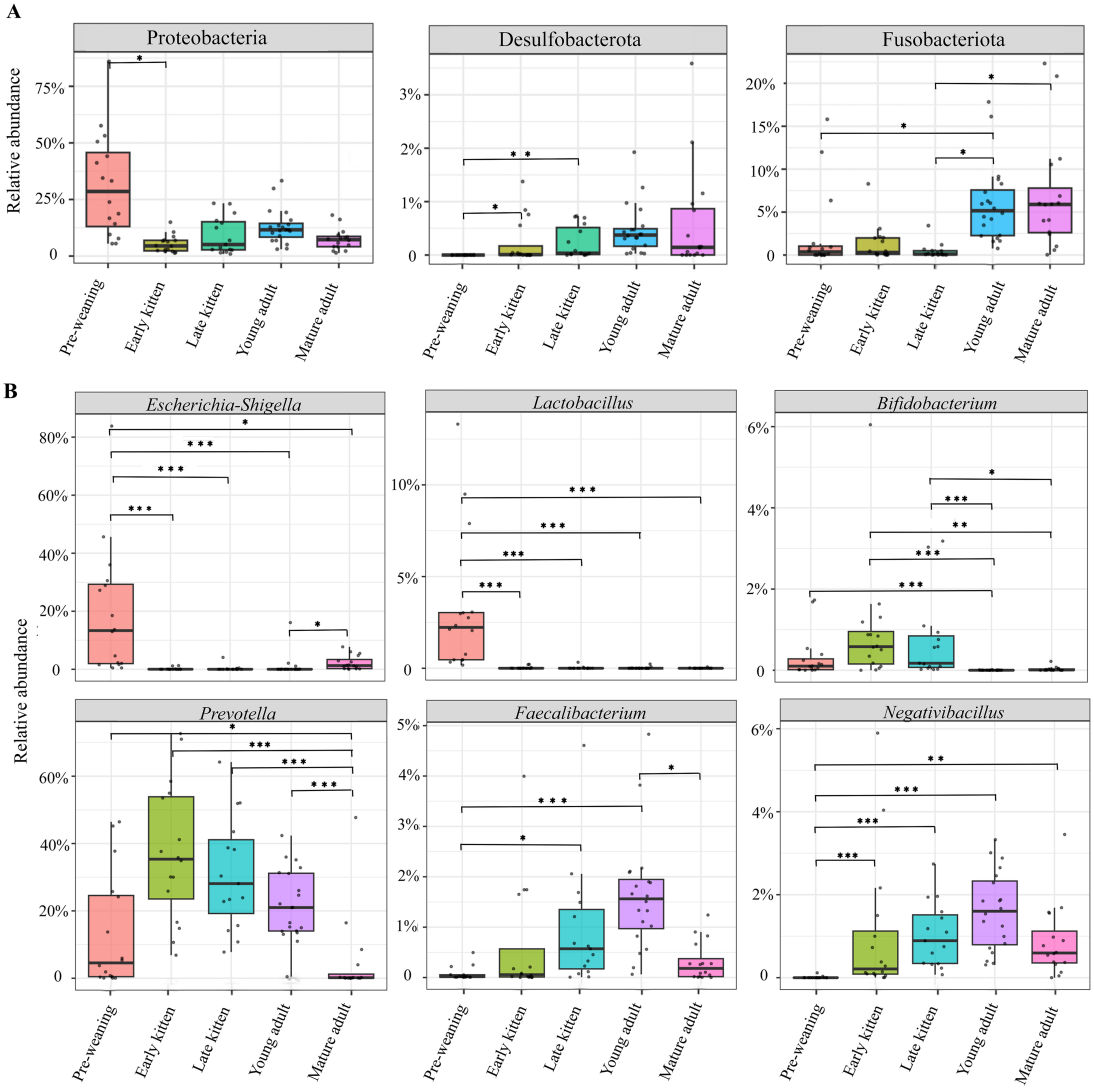
Comparative analysis of fecal microbiota composition across age-defined groups in cats. **(A)** Relative abundance of major bacterial phyla exhibiting significant variations among groups based on ANCOM-BC2 analysis. **(B)** Relative abundance of representative bacterial genera identified by ANCOM-BC2 analysis. Box plots represent the median (horizontal line), and the first and third quartiles (box limits), with whiskers extending to 1.5 times the interquartile range. Individual data points are shown as overlaid jitter points. Asterisks indicate significant differences between groups based on ANCOM-BC2 differential abundance testing (*q < 0.05, **q < 0.01, ***q < 0.001). The five groups are defined by age: pre-weaning, early kitten, late kitten, young adult, and mature adult.

Differential abundance analysis across the five age-defined groups was performed using the ANCOM-BC2 method. At the phylum level, nine bacterial phyla showed significant differences among groups (FDR-adjusted *q* < 0.05; Supplementary Table S3). For clarity of presentation, three representative taxa that exhibited significant pairwise differences between groups were selected for visualization and detailed analysis, including Proteobacteria, Fusobacteriota, and Desulfobacterota (Figure 4A; Supplementary Table S4). Among these taxa, Proteobacteria was significantly enriched in the Pre-weaning stage compared with the Early kitten stages (*q* < 0.05), In contrast, Desulfobacterota showed significantly higher abundance in both the Early kitten and Late kitten stages relative to Pre-weaning cats (*q* < 0.05). Furthermore, Fusobacteriota showed significantly higher abundance in both Young adult and Mature adult cats compared with the Late kitten group (*q* < 0.05), indicating clear variation in phylum-level profiles across age-defined groups. Similar patterns were observed after excluding the Mature adult group, with the enrichment of Proteobacteria in Pre-weaning cats and the increasing trends of Fusobacteriota and Desulfobacterota across subsequent stages remaining statistically significant.

### 3.4 Variations in key bacterial genera across age-defined groups

At the genus level, the top 10 dominant bacteria in each group in terms of relative abundance included *Prevotella*, *Bacteroides*, *Megamonas*, *Megasphaera*, *Anaerobiospirillum*, *Escherichia*-*Shigella*, *Alloprevotella*, *Campylobacter*, *Cetobacterium*, and *Phascolarctobacterium* (Figure 3B; Supplementary Table S2).

A total of 102 genera showed significant differences among the five age-defined groups according to the ANCOM-BC2 analysis (FDR-adjusted *q* < 0.05; Supplementary Table S5). For visualization, representative genera showing significant differential abundance and consistent pairwise differences between groups were selected for display (Figure 4B; Supplementary Table S6). Notably, *Escherichia-Shigella* and *Lactobacillus* were significantly enriched in Pre-weaning kittens (ANCOM-BC2, *q* < 0.001). In contrast, *Negativibacillus* significantly increased after weaning (*q* < 0.001). The abundance of *Bifidobacterium* was significantly higher in both Early and Late kitten stages than in adults, with a Log Fold Change ranging from 3.17 to 5.40 in pairwise comparisons between kitten and adult groups (*q* < 0.05). *Faecalibacterium* showed an upward trend across the developmental groups, with significantly higher levels maintained in Young adult cats compared to the Preweaning kittens (*q* < 0.001). Similarly, *Prevotella* levels were significantly reduced in Mature adult cats relative to younger groups (*q* < 0.001). Notably, while *Escherichia-Shigella* declined after weaning, it exhibited a significant increase in Mature adult cats compared with the Young adult group (*q* < 0.05). These results collectively indicate that the feline gut microbiota exhibits distinct group-specific compositional differences across the defined groups, with unique taxonomic signatures identified in the pre-weaning, kitten, and adult groups. Sensitivity analyses excluding the Mature adult group confirmed that these genus-level patterns remained largely preserved among the younger groups, although effect sizes were modestly attenuated (Supplementary Tables S7, S8).

Furthermore, functional profiles inferred using PICRUSt2 indicated potential differences across groups. However, as these predictions are based on 16S rRNA gene data rather than direct functional measurements, they should be interpreted cautiously and considered hypothesis-generating. Detailed results, including correlations between specific genera and KEGG pathways, are provided in Supplementary Figure S3.

## 4 Discussion

The composition of gut microbiota has been reported to vary across life stages in mammals. However, in felines, few studies have utilized molecular techniques to characterize the variations in gut microbiota composition across life stages. In this study, based on 16S rRNA gene sequencing, we characterized the gut microbiota across five age-defined groups, from Pre-weaning to Mature adult cats. Our results showed that alpha-diversity, as well as community composition and structure, differed significantly among these groups under varying environmental conditions. Importantly, these differences should be interpreted as cross-sectional, group-level patterns that may reflect the combined influences of host development, environmental exposure, and dietary variation, rather than age-related biological effects alone. Sensitivity analyses excluding the Mature adult group showed that several key taxonomic differences remained statistically significant across the remaining groups, although effect sizes were modestly attenuated and residual confounding cannot be excluded.

Previous research supports that the majority of bacteria found in the feline gastrointestinal tract belong to five distinct phyla: Firmicutes, Bacteroidota, Fusobacteria, Proteobacteria, and Actinobacteria (19–21). In our study, the three predominant bacterial phyla identified were Bacteroidota, Firmicutes, and Proteobacteria, followed by Campylobacterota, Fusobacteria, and Actinobacteria. Notably, the taxonomic and functional divergence between Campylobacterota and Proteobacteria merits attention. Although both phyla comprise Gram-negative bacteria historically classified within Proteobacteria (22, 23). Campylobacterota was reclassified as a distinct phylum in 2019 based on conserved signature and phylogenomic analyses (24). In our result, Proteobacteria dominated the gut microbiota of Pre-weaning kittens (Figure 3A), a pattern that aligns with observations of this taxon as a dominant early colonizer in young mammals. Consistent with this, metagenomic DNA sequence-based studies on canine and feline gut microbiota by Moon et al. (25) indicated that Proteobacteria had a relatively high early abundance in the intestines of puppies and kittens, and their metabolic strategies such as utilizing simple carbon sources, proteins and consuming oxygen were highly compatible with the immature intestinal microenvironment. While functional profiling (Supplementary Figure S3) suggested associations between these early colonizers and predicted metabolic pathways, such as phosphonate metabolism, these 16S-derived inferences remain strictly hypothesis-generating. These associations should be interpreted cautiously and do not imply direct functional activity or mechanistic relationships. This observation is consistent with findings in studies on human infants, where Proteobacteria act as early colonizers (26, 27). For instance, a study investigating the establishment of gut microbiota in 1-year-old infants found that those exclusively breastfed until 6 months were mainly colonized by *Lactobacillaceae* and *Enterobacteriaceae*—a major family within Proteobacteria (28). Notably, in humans, the infant gut has a high abundance of ARGs compared to adults even when infants have not been exposed to antibiotics. The high abundance of Gammaproteobacteria in the infant gut has been hypothesized to explain the higher levels of ARGs, as Gammaproteobacteria often carry several resistance genes. Whether a similar association exists in Pre-weaning kittens remains unclear and requires further investigation. Interestingly, *Escherichia-Shigella* showed a secondary increase in Mature adult cats, which may reflect microbiota instability or environmental influences in later life stages, although the underlying mechanisms remain unclear.

At the genus level, we observed that *Prevotella* was the dominant genus in Early kittens (post-weaning), whereas its relative abundance was significantly reduced in Mature adult cats compared with earlier stages (*q* < 0.001). It has been reported that *Prevotella* is a metabolically versatile microbe, capable of processing a wide range of proteins and polysaccharides, and one of its fermentation products is propionate, a type of short-chain fatty acid (29, 30). Based on PICRUSt2 functional prediction, the relative abundance of *Prevotella* showed a negative correlation with predicted pathways related to phosphonate and phosphinate metabolism. However, as these inferences are based on predictive approaches, they should be interpreted cautiously and regarded as hypothesis-generating rather than direct functional evidence. In contrast, *Bacteroides*, another major member of the Bacteroidota phylum, showed a non-significant increasing trend in Mature adult cats relative to younger groups (*q* > 0.05). In many mammalian models, both *Prevotella* and *Bacteroides* are highly sensitive to dietary macronutrient profiles, particularly the carbohydrate-to-protein ratio and fiber content (31, 32). Therefore, the reduced abundance of *Prevotella* and the tendency toward increased *Bacteroides* observed in the Mature adult group (client-owned cats) may reflect group-specific dietary variation rather than chronological aging alone. Given the lack of detailed dietary metadata (e.g., dry versus wet food ratios or specific formulations), dietary heterogeneity remains a plausible contributing factor that cannot be disentangled from age in the present study. Importantly, sensitivity analyses excluding the Mature adult group showed that several genus-level patterns across earlier life stages remained statistically consistent, although residual confounding cannot be excluded. Additionally, *Faecalibacterium* was more abundant in Young adult cats compared with earlier stages, which may indicate features of a more stabilized adult-associated microbiota. However, these observations should be interpreted as associations rather than causal age-related effects. The relative contributions of host development, diet, and environment to these observed patterns warrant further investigation through controlled longitudinal studies.

*Bifidobacterium* and *Lactobacillus* are the main probiotic genera in felines (33), playing important roles in immune function and digestive health (34, 35). Our results revealed that *Lactobacillus* was enriched in the fecal microbiota of Pre-weaning kittens but declined significantly in subsequent groups, whereas *Bifidobacterium* was significantly more abundant in both Early and Late kitten stages than in adults (*q* < 0.05). Such variations suggest distinct, group-specific distribution patterns of these genera across age-defined groups. *Lactobacillus* has been reported to be associated with milk digestion and gut barrier function during the milk-dominated Pre-weaning phase. Following the dietary transition to solid food, the higher abundance of *Bifidobacterium* along with *Prevotella* observed in post-weaning kittens may reflect group-specific variations associated with dietary transition between groups. Given that kittens undergo substantial biological and environmental changes during early life, these observations may provide a basis for future investigation into microbiota-targeted strategies to support intestinal health during these complex developmental periods.

This study has several limitations that warrant cautious interpretation. Primarily, as a cross-sectional study, the observed differences across age-defined groups represent group-specific snapshots rather than longitudinal trajectories; thus, within-individual microbial succession could not be determined. A fundamental limitation is the structural confounding between age, housing, and diet: younger cats were sourced from catteries with standardized regimens, whereas Mature adults were client-owned pets with diverse dietary backgrounds. Consequently, the observed differences in key taxa—such as *Prevotella*, *Bacteroides*, and *Fusobacteriota*—cannot be solely attributed to age, as environmental and nutritional factors may significantly confound these variations. Due to the lack of detailed dietary metadata for household cats, we were unable to include these factors as covariates or perform stratified analyses to disentangle their relative contributions. However, a sensitivity analysis excluding the Mature adult group was conducted to partially assess the robustness of the observed patterns. Furthermore, the limited statistical power associated with our relatively small sample sizes (*n* = 15–20 cats per group) is a significant concern. Given the high between-individual variability typical in microbiome research, coupled with the aforementioned confounding variables, there is an increased risk of spurious associations. Additionally, as stated previously, breed influences on the microbiome cannot be separated from age effects in this dataset. Finally, our reliance on 16S rRNA sequencing and PICRUSt2-based functional predictions limits taxonomic resolution to the genus level and provides inferred rather than directly measured metabolic profiles, meaning these functional insights remain strictly hypothesis-generating. Other potential confounders—including sex, stress, neuter status, breed, and small-scale cattery-specific effects—were not fully controlled. Notably, breed may independently influence gut microbiome composition but could not be disentangled from age in this study design. Additionally, the recruitment from a single geographic region and the modest sample size may limit the generalizability of these findings. Future controlled longitudinal studies with standardized diets across all life stages are necessary to validate these observations and disentangle the relative contributions of age, diet, and environment.

## 5 Conclusion

In conclusion, this study describes distinct compositional and structural differences in the feline gut microbiota across age-defined groups, ranging from Pre-weaning kittens to Mature adult cats. These observed differences likely reflect the combined influences of host development, diet, and living environment, rather than chronological age alone. Sensitivity analyses indicated that key microbial patterns among the younger age groups remained consistent, although residual confounding cannot be excluded. While our findings provide a descriptive framework for understanding group-specific microbiota variation, further controlled longitudinal studies are required to fully disentangle these intertwining factors. Ultimately, this baseline description provides a reference framework for future hypothesis-driven and controlled investigations of the feline gut microbiome. Incorporating these microbial insights into personalized interventions— such as precision probiotics (36) and stage-specific nutritional strategies tailored to individual dietary and lifestyle factors (e.g., sex, age, and lifestyle) offers a hypothetical framework that requires rigorous validation in controlled, longitudinal settings before clinical application.

## Data Availability

The datasets presented in this study can be found in online repositories. The names of the repository/repositories and accession number(s) can be found below: National Microbiology Data Center (NMDC), NMDC40110805–NMDC40110887.
