# Supplementary material for: Cross-sectional analysis of feline gut microbiota reveals differences across age-defined groups under varying environments
Source: Front Vet Sci. 2026 May 12;13:1775401. doi: 10.3389/fvets.2026.1775401 (PMC13205103; doi:10.3389/fvets.2026.1775401)
Supplement: Supplementary file 1 [file Data_Sheet_1.docx]

Supplementary Figures for:

**Cross-Sectional Analysis of Feline Gut Microbiota Reveals Differences Across Age-Defined Groups Under Varying Environments**





**Supplementary Figure 1.** Alpha-diversity (Chao1 and Shannon indices) of the feline gut microbiota across four age-defined groups in the sensitivity analysis after exclusion of the Mature adult group. Differences across groups were tested using the Kruskal–Wallis test (overall p < 0.001), followed by Dunn’s *post-hoc* test with FDR correction for pairwise comparisons (**q* < 0.05, ****q* < 0.001).

**
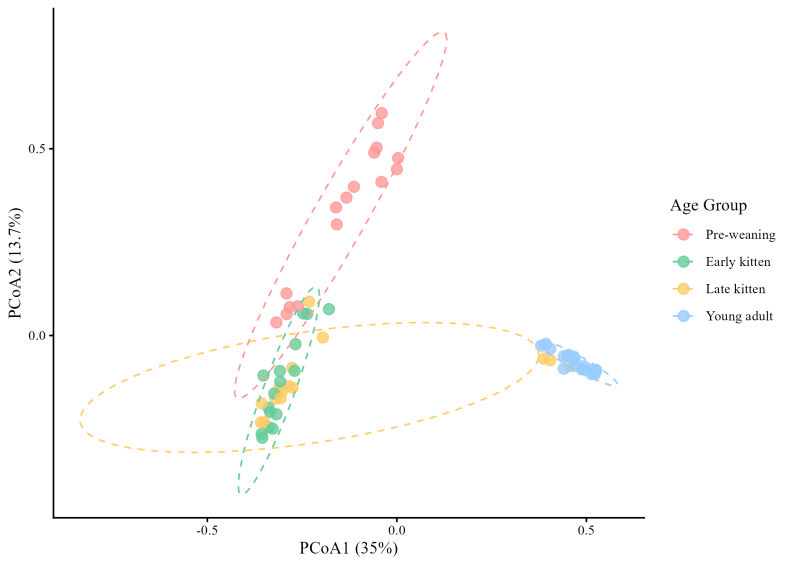
**

**Supplementary Figure 2.** Principal Coordinate Analysis (PCoA) based on Bray–Curtis dissimilarity showing beta-diversity differences across four age-defined groups in sensitivity analysis after exclusion of the Mature adult group. Statistical significance was assessed using PERMANOVA (*R*² = 0.48, *p* = 0.001).

**
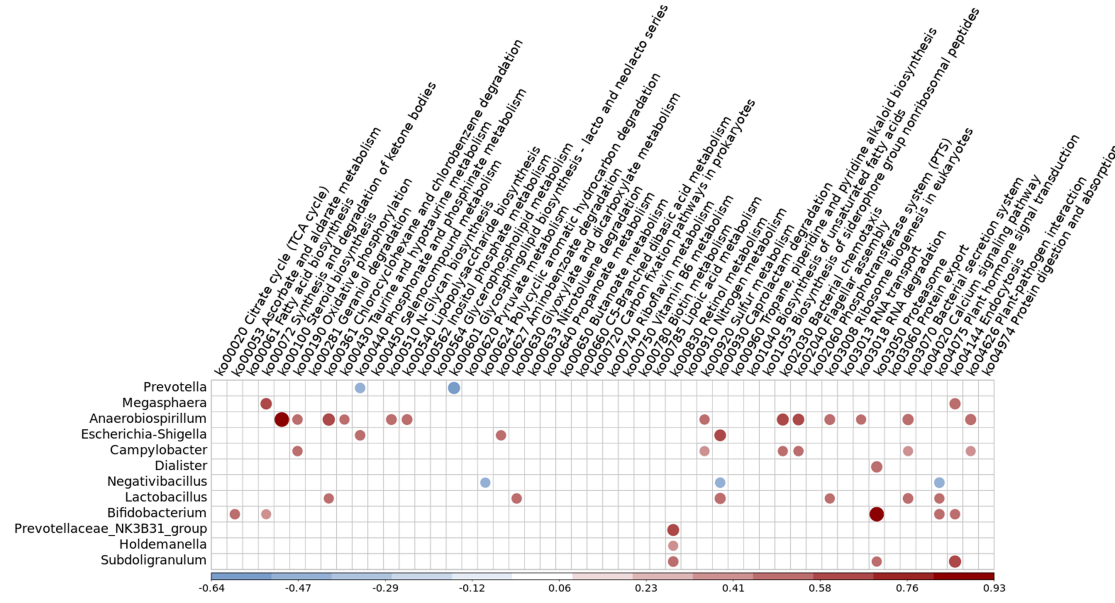
**

**Supplementary Figure 3.** Heatmap of Spearman rank correlation analysis (*p* < 0.05) between differentially abundant bacterial genera and predicted functional pathways (inferred by PICRUSt2). Red and blue indicate positive and negative correlations, respectively, between the relative abundance of bacterial genera and the predicted abundance of these pathways.

Spearman’s rank correlation analysis indicated multiple associations between differentially abundant genera and predicted functional pathways. Genera enriched in younger age-defined groups, particularly the Pre-weaning group, generally showed positive correlations with inferred metabolic and environmental adaptation-related pathways, whereas some genera, such as *Prevotella*, exhibited negative associations with specific predicted functions. These results are derived from PICRUSt2-based functional predictions and should be interpreted strictly as hypothesis-generating exploratory findings.
